# Exploring the differences in atmospheric mesoscale kinetic energy spectra between AI based and physics based models

**DOI:** 10.1038/s41598-025-99815-x

**Published:** 2025-05-03

**Authors:** Zongheng Li, Jun Peng, Lifeng Zhang, Hanyan Wu, Wei Zhang, Juan Zhu

**Affiliations:** 1 College of Meteorology and Oceanography, National University of Defense Technology, Changsha, China; 2 Zhangzhou Base Preparation Office, National Ocean Technology Center, Xiamen, China

**Keywords:** Atmospheric dynamics, Fluid dynamics, Atmospheric dynamics

## Abstract

It is an urgent need to understand the ability of current artificial intelligence (AI) models in simulating atmospheric mesoscale aspects. This paper compares mesoscale kinetic energy spectra from an 11-day experiment simulated by a novel AI-based model (Pangu) and a physics-based model (MPAS), using ERA5 reanalysis as a reference. Based on the commonly used evaluation metrics of latitude weighted root mean square error (RMSE) and anomaly correlation coefficient (ACC), the AI-based model has better short to medium-range weather forecasting skill compared to the physics-based model. However, the AI-based model cannot replicate the mesoscale − 5/3 spectral slope and underestimates the mesoscale energy at wavelength smaller than 1000 km. As altitude increases and scale decreases, the deviation of the AI-based model from the reanalysis significantly increases. These features prove that the AI-based model has the lower effective resolution compared to the physics-based model with the close nominal resolution. Compared to the physics-based simulations, AI-based model has stronger downscale energy flux at larger mesoscales, which is dominated by divergent kinetic energy flux. But it rapidly becomes the weakest at smaller mesoscales. The diagnosed vertical velocity of AI-based model and its related budget terms are closest to those of the reanalysis at large scales. Overall, the AI-based model Pangu shows closer agreement with ERA5 at large scales, likely due to its use of the latter as training data, but significantly underestimates mesoscale kinetic energy compared to the physics-based model MPAS. Note that these findings are specific to the models and configurations used and should be interpreted with caution.

## Introduction

In recent years, a novel approach to weather forecasting using artificial intelligence (AI) has emerged. It usually employs deep neural networks trained on historical atmospheric data from reanalysis datasets^1–3^. By analyzing past atmospheric variations, neural networks can predict future states without relying on explicit representation of physical laws. Recent findings indicate that these AI-based models can achieve forecast accuracy that is comparable to, or even surpass, that of traditional numerical models^2^.

However, it seems that AI-based models have not met people’s expectations in their performance of certain phenomena. For example, a quantitative evaluation case study of Storm Ciarán is carried among different AI-based models and physics-based models and find that all AI-based models underestimate the peak amplitude of winds associated with the storm^4^. In addition, the AI-based model (Pangu) fails to replicate the rapid initial error growth rates, suggesting erroneously unlimited atmospheric predictability^5^. Thus, the dynamic mechanism behind the simulation features of AI-based models is a question worthy of in-depth research.

The kinetic energy (KE) spectrum is a fundamental physical property of the atmosphere. The wavenumber spectrum of the global atmosphere allows us to understand the energy distribution across spatial scales. The corresponding energy spectral budget reveals energy sources/sinks and energy dissipation of atmospheric motion at different spatial scales^6^. The structure of atmospheric KE spectra follows a canonical pattern, as evidenced by both simulations and observations^7–9^. The spectrum exhibits a logarithmic power-law relationship with scale. Analyses of atmospheric KE spectrum reveal a -3 slope at synoptic scales, transitioning to a -5/3 slope within the mesoscales^8^. The dynamical origins of the mesoscale spectral slope are still controversial^10^. To date, the global mesoscale spectra show significant inconsistencies among different global reanalysis datasets^11^. Global mesoscale kinetic energy is significantly affected by convection, which show considerable variation across reanalyses^11,12^. The atmospheric kinetic energy spectrum is widely used in the development and evaluation of numerical models^9,13–15^. The accurate representation of this canonical spectral behavior is often used to validate the design, configuration, and performance of numerical weather prediction (NWP) models^9,13^. Furthermore, atmospheric energy spectra are crucial for understanding atmospheric predictability across scales, yet it remains poorly understood^16,17^.

In this study, we examine the capability of an AI-based model (Pangu) to simulate the mesoscale kinetic energy by comparing with the Fifth generation of ECMWF atmospheric reanalysis of the global climate (ERA5)^18^ and simulations from a numerical prediction model based on physics-based discretization (Model for Prediction Across Scales^9^, MPAS). Moreover, it is crucial to explain the underlying cause of the power-law behavior observed in the kinetic energy spectrum produced by the Pangu model, particularly at mesoscales, which is also the purpose of the paper.

We selected 100 hPa to represent the lower stratosphere and 300 hPa to represent the upper troposphere. The motivation for such choice was to avoid selecting a level that spans the tropopause and to minimize the impact of terrain as much as possible. In addition, we select 850 hPa to represent the lower troposphere. Since the lack of observations, ERA5 is considered as the benchmark in the study, and simulation deviations from ERA5 are considered as model errors. Although ERA5 is widely used as a high-quality reanalysis product, it is important to note that it is still an estimate with inherent uncertainties. The spectral characteristics of ERA5 may not represent the true atmospheric dynamics perfectly. Therefore, while ERA5 serves as a useful benchmark for our analysis, our conclusions should be interpreted with caution.

In order to gain more physical insights, the KE is split into rotational and divergent components, roughly corresponding to vortices and inertia–gravity waves contributions, and the differences on the multiscale dynamics of modelling atmosphere are further revealed by diagnosing the spectral rotational and divergent kinetic energy budget, with focus on energy cascade, i.e., the energy transfer among different scales.

## Dataset, models and methods

### Analysis product ERA5

ERA5 is used as a measure of forecasts’ truth because the AI models all used ERA5 as their training data. Hence comparison with ERA5 indicates the skillfulness of these models relative to the best possible forecast given their training data. The ERA5 system used a T639 spectral model (~ 0.28° grid) and an N320 reduced Gaussian grid, and the analysis increment was obtained by running three successive inner loops at resolutions of T95, T159, and T255 to minimize the cost function^18^.

### AI-based model Pangu

This study uses “Pangu-Weather”^2^, a data-driven AI-based model as a representative, which produces slightly better forecasts than the leading operational weather prediction model (Integrated Forecast System, IFS). Pangu consists of a 3D^19^ deep neural network trained with 39 years of ERA5 data, and it provides forecasts for 4 different time steps (1 hr, 3 hr, 6 hr, 24 hr). Longer time steps produce better forecasts, and a “hierarchical temporal aggregation” technique is used to produce forecasts every 3 hours. The model state of Pangu includes 13 pressure levels and 9 variables (5 upper-air and 4 surface variables) on a regular 0.25° latitude-longitude grid.

### Physics-based model MPAS

This study uses the MPAS-A version 7.3 global model from the National Center for Atmospheric Research (NCAR). The model has a top at 30 km, consisting of 55 vertical levels with a gravity wave absorbing layer in the top 8 km^20^. In this paper, the horizontal grid spacing is set to 30 km and 15 km. The former is close to the resolution of ERA5, and the latter is a bit larger than that of current global operational NWP models. It resolves some mesoscale regimes but still parameterizes convection and gravity waves. The dynamics and physics time steps are both set to 180 s–90 s, and a fourth-order horizontal filter is applied. The physics schemes include the convection parameterization scheme New Tiedtke^21^, the cloud microphysics scheme WSM6^22^, the land surface model Noah^23^, the Yonsei University (YSU)^24^, Monin-Obukhov^25^, and the Rapid Radiative Transfer Model (RRTMG)^26^.

### Experiments

In this study, three different experiments were designed, the Pangu test, the MPAS test with a horizontal resolution of 30 km, and the MPAS test with a horizontal resolution of 15 km, which were denoted as Pangu, MPAS30, and MPAS15, respectively. The Pangu test initialized the Pangu model using ERA5 reanalysis data and made an 11-day forecast starting at 0000 UTC on July 10, 2021, with results output every 3 h. The iterative strategy uses Pangu’s hierarchical temporal aggregation strategy so that the forecast at each moment passes the least number of times. The MPAS tests also use ERA5 reanalysis data to initialize the MPAS model. The simulation started at 00:00 UTC on July 10th, 2021. We generated 11-day simulations with outputs every three hours, and we believe that the fields in these simulations will be fully spun up within one day.

### Metrics

The main metrics to evaluate the prediction performance are latitude-weighted root-mean-square error (RMSE) and latitude-weighted anomaly correlation coefficient (ACC)^27^, which are calculated as follows:

1 $$\:RMSE=\sqrt{\frac{1}{{N}_{lat}{N}_{lon}}{\sum\:}_{j}^{{N}_{lat}}{\sum\:}_{i}^{{N}_{lon}}L\left(j\right)({forecast}_{i,j}-{truth}_{i,j}{)}^{2}}$$

Among them, $\:forcast$ is the model forecast, which $\:truth$ is the ERA5 true value. $\:L\left(j\right)$ is the latitude weighting factor for the latitude at the $\:j$th latitude index:

2 $$\:L\left(j\right)=\frac{{cos}\left(lat\left(j\right)\right)}{\frac{1}{{N}_{lat}}{\sum\:}_{j}^{{N}_{lat}}{cos}\left(lat\left(j\right)\right)}$$

In addition, we use the latitude-weighted anomaly correlation coefficient (ACC) for the assessment. *N*_*lat*_ and *N*_*lon*_ represent the number of grid points in the latitudinal and meridional directions respectively.

3 $$\:ACC=\frac{{\sum\:}_{i,j}L\left(j\right){forecast}_{i,j}^{{\prime\:}}{true}_{i,j}^{{\prime\:}}}{\sqrt{{\sum\:}_{i,j}L\left(j\right){forecast}_{i,j}^{{\prime\:}2}}\sqrt{{\sum\:}_{i,j}L\left(j\right){true}_{i,j}^{{\prime\:}2}}}$$

where the superscript ′ indicates the difference from the climatic average. The climatic regime here is defined as:

4 $$\:{\text{C}\text{limatology}}_{i,j}=\frac{1}{{N}_{time}}\sum\:{t}_{i,j}$$

Specifically, Climatology represents the global atmosphere of 1.5° × 1.5° daily calculated the climatology (1989–2008) using ERA5 reanalysis data., which are also interpolated to a linear Gaussian grid of N320.

### The KR and KD spectral budget

Before calculating the spectra, all the fields were interpolated into linear Gaussian grid N320 through Climate Data Operators (CDO). The calculation of kinetic energy spectra and spectral budget is based on our previous research^15,28^. The total wavenumber is the order of spherical harmonics ($\:\varvec{l}$, dimensionless). The wavelength is estimated by $\:2\varvec{\pi\:}\varvec{R}/\varvec{l}$, where $\:\varvec{R}$ is the radius of the earth. Although we have diagnosed the vertical velocities, they are still not reliable enough to compute the spectral budget. Thus, we only conduct the spectral budget without the pressure vertical velocity $\:\varvec{\omega\:}$ (i.e., the vertical velocity in the pressure vertical coordinate $\:\varvec{d}\varvec{p}/\varvec{d}\varvec{t}$). Then, the spectral budget equation only retains terms independent of vertical velocity. The details of the budget terms are as follows:

5 $$T_{D}^{{lm}}= - \left[ {{{({{\varvec{u}}_D},\nabla {E_K})}_{lm}}+\frac{{{{({\varvec{u}},{\varvec{u}}\delta )}_{lm}}}}{2}} \right] - \frac{{\left[ {{{({{\varvec{u}}_D},f{{\varvec{e}}_z} \times {\varvec{u}})}_{lm}}+{{({\varvec{u}},f{{\varvec{e}}_z} \times {{\varvec{u}}_D})}_{lm}}} \right]}}{2} - \frac{{\left[ {{{({{\varvec{u}}_D},\zeta {{\varvec{e}}_z} \times {\varvec{u}})}_{lm}}+{{({\varvec{u}},\zeta {{\varvec{e}}_z} \times {{\varvec{u}}_D})}_{lm}}} \right]}}{2}$$

6 $$T_{R}^{{lm}}= - \frac{{\left[ {{{({{\varvec{u}}_R},f{{\varvec{e}}_z} \times {\varvec{u}})}_{lm}}+{{({\varvec{u}},f{{\varvec{e}}_z} \times {{\varvec{u}}_R})}_{lm}}} \right]}}{2} - \frac{{\left[ {{{({{\varvec{u}}_R},\zeta {{\varvec{e}}_z} \times {\varvec{u}})}_{lm}}+{{({\varvec{u}},\zeta {{\varvec{e}}_z} \times {{\varvec{u}}_R})}_{lm}}} \right]}}{2}$$

7 $$C_{{D \to R}}^{{lm}}=+\frac{{\left[ {{{({{\varvec{u}}_D},f{{\varvec{e}}_z} \times {{\varvec{u}}_R})}_{lm}} - {{({{\varvec{u}}_R},f{{\varvec{e}}_z} \times {{\varvec{u}}_D})}_{lm}}} \right]}}{2}+\frac{{\left[ {{{({{\varvec{u}}_D},\zeta {{\varvec{e}}_z} \times {{\varvec{u}}_R})}_{lm}} - {{({{\varvec{u}}_R},\zeta {{\varvec{e}}_z} \times {{\varvec{u}}_D})}_{lm}}} \right]}}{2}$$

Here, $\:{T}_{D}^{lm}$and $\:{T}_{R}^{lm}$denote the spectral divergent and rotational kinetic energy transfer terms without considering the vertical motion, respectively. $\:{\varvec{u}}_{R}$ and $\:{\varvec{u}}_{D}$ denote rotational and divergent components of horizontal velocity, respectively. $\:\zeta\:$ and $\:\delta\:$ denote relative vorticity and horizontal divergence, respectively. $\:{\varvec{e}}_{z}$ is the vertical (upward) unit vector, $\:f\:$is the Coriolis parameter. The vertically integrated spectral fluxes and accumulated spectral conversion can be written as follows:

8 $$\:{\varPi\:}_{R}\left[l\right]={\sum\:}_{l\ge\:n}{\int\:}_{{p}_{t}}^{{p}_{b}}\frac{dp}{g}{\sum\:}_{\left|m\right|\le\:n}{T}_{R}^{nm}$$

9 $$\:{\varPi\:}_{D}\left[l\right]={\sum\:}_{l\ge\:n}{\int\:}_{{p}_{t}}^{{p}_{b}}\frac{dp}{g}{\sum\:}_{\left|m\right|\le\:n}{T}_{D}^{nm}$$

10 $$\:{\mathcal{C}}_{D\to\:R}\left[l\right]={\sum\:}_{l\ge\:n}{\int\:}_{{p}_{t}}^{{p}_{b}}\frac{dp}{g}{\sum\:}_{\left|m\right|\le\:n}{C}_{D\to\:R}^{nm}$$.

The calculation is divided into three steps: Step 1, the spectral transfers summed over all zonal wavenumbers $\:m$ at a given total wavenumber $\:l$; Step 2, vertically integrated between two pressure levels $\:{p}_{b}$ and $\:{p}_{t}$, and divided by $\:g$; Step 3, summed over all total wavenumbers greater than or equal to $\:l$.

When considering the pressure vertical velocity $\:\omega\:$, the vertical flux and APE conversion can be expressed as

11 $$F_{{D \uparrow }}^{{lm}}= - \left[ {{{\left( {\omega ,~\Phi } \right)}_{lm}}+~\frac{{{{\left( {{\varvec{u}},\omega {\varvec{u}}} \right)}_{lm}}}}{2}} \right]$$

12 $$\:{C}_{AP\to\:D}^{lm}=-(\omega\:,-\frac{RT}{p}{)}_{lm}$$

where Φ is the geopotential, *T* is the temperature and *R* is the gas content. The accumulated forms are as follows

13 $$\:{\mathcal{C}}_{AP\to\:D}\left[l\right]={\int\:}_{{p}_{t}}^{{p}_{b}}\frac{dp}{g}{\sum\:}_{n\ge\:l}{\sum\:}_{\left|m\right|\le\:n}{C}_{AP\to\:D}^{nm}$$

14 $$\:{\mathcal{F}}_{D\uparrow\:}\left[l\right]={\sum\:}_{n\ge\:l}{\sum\:}_{\left|m\right|\le\:n}\left({F}_{D\uparrow\:}^{nm}\left[{p}_{b}\right]-{F}_{D\uparrow\:}^{nm}\left[{p}_{t}\right]\right)$$

### Diagnostics of the pressure vertical velocity

The stronger vertical velocities are usually associated with stronger and deeper convection and larger gravity wave momentum flux^29^. Unfortunately, the vertical velocity is not included in the output of Pangu. Thus, we try to diagnose the pressure vertical velocity $\:\varvec{\omega\:}$ through the horizontal wind based on the continuity equation. In pressure coordinates, the continuity equation is naturally simplified into an incompressible form, and thus the vertical pressure can be obtained by the vertical integration of the horizontal divergence. That is, integrating the continuity equation from the model top ($\:\varvec{p}=0$) to an isobaric level $\:\varvec{p}$ and using the upper boundary condition $\:\varvec{\omega\:}\left(0\right)=0$ gives the $\:\varvec{\omega\:}$ at $\:\varvec{p}$:

15 $$\:\omega\:\left(p\right)=-{\int\:}_{0}^{p}\left(\nabla\:\cdot\:\varvec{u}\right)dp$$

where $\:\varvec{u}$ presents the horizontal wind field. Note that the limited vertical resolution results in diagnosing errors.

## Results

### A simple evaluation of the predictive ability of the models

**Fig. 1:**
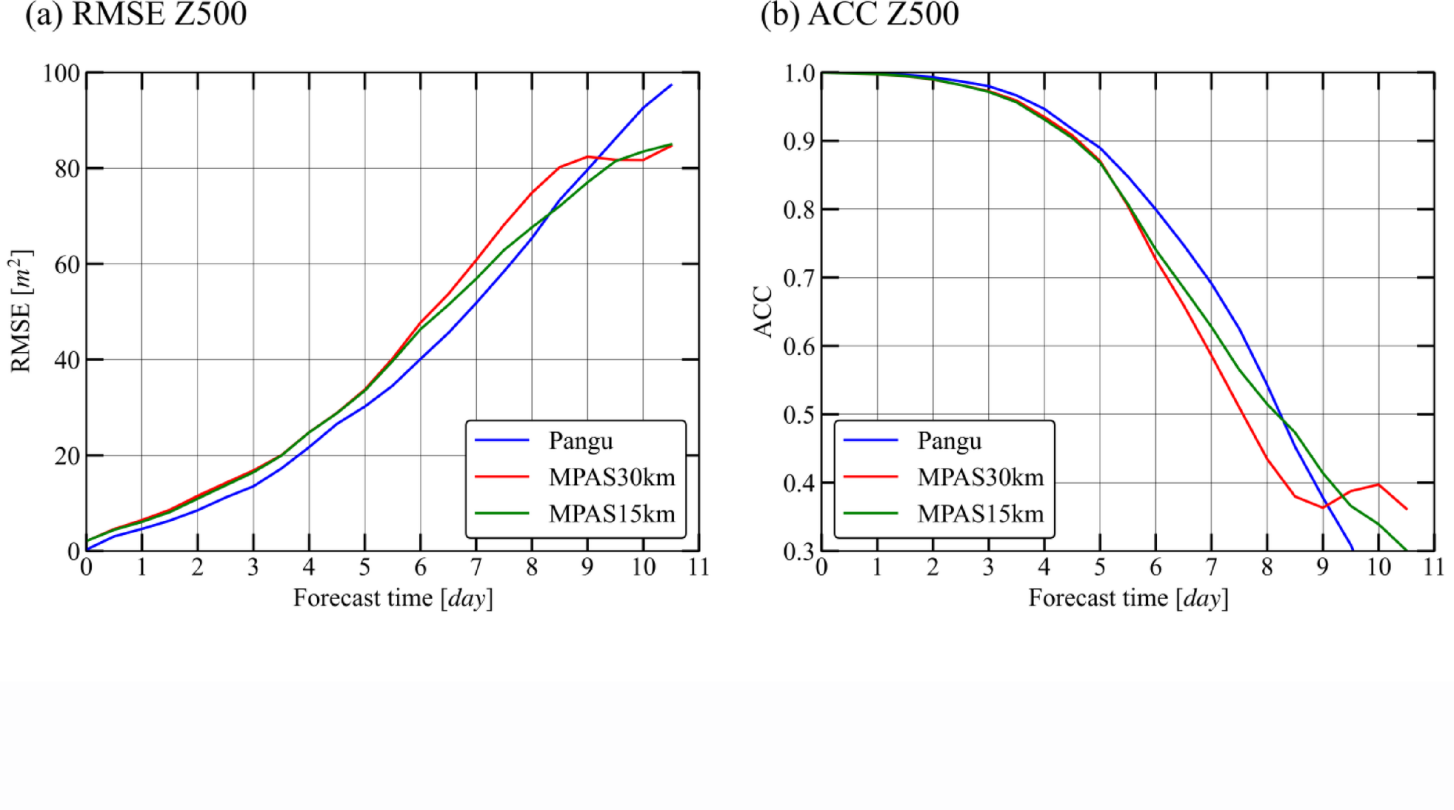
Evaluation of the models’ accuracy in deterministic forecasts against ERA5. (**a**) the latitude-weighted RMSE; lower is better. (**b**) the latitude-weighted ACC; higher is better. Z500 indicates the height at 500 hPa.

The latitude-weighted root-mean-square error (RMSE) and anomaly correlation coefficient (ACC) are two common metrics to evaluate the prediction performance^27^, according to Eqs. (1–4). The 500 hPa height (Z500) is often used to evaluate prediction capabilities. From these two metrics, Pangu’s predictive ability is superior to the MPAS models with two resolutions (Fig. 1). Note that the MPAS model only runs the atmospheric module without external forcing processes such as sea surface temperature variations, which can improve the accuracy of MPAS model predictions. In the first 8 days, the RMSE of the Pangu was always smaller than that of the MPAS15 or MPAS30, and then the RMSE of the Pangu exceeded that of the MPAS. Consistently, the ACC of the Pangu simulation was higher than that of MPAS15 and MPAS30 in the first 8 days, after that time the ACC of Pangu was lower. When ACC is equal to 0.5, the prediction period of Pangu are 15.5 h longer than that of MPAS30. Although there are significant uncertainties in the long-term forecast, it is clear that in later stages the results of the numerical models are better than those of the artificial intelligence model, which may be due to the lack of physical constraints in the Pangu model. This indicates that with identical initial fields, the AI model demonstrates more accurate short- to-medium-term forecasting capabilities than the physics-based models. However, there is a question that arises whether this improvement in ability is due to better analysis of mesoscale processes (such as storms and typhoons) or better description of mesoscale energy.

### Dynamical evaluation of models through kinetic energy spectra

All spectra herein are computed by averaging spectra computed over the 10-day period at 3 hourly intervals from 0000 UTC 11 July to 1800 UTC 20 July 2021. The three pressure levels of 850 hPa, 300 hPa and 100 hPa are selected to represent the lower troposphere, upper troposphere and lower stratosphere, respectively.

**Fig. 2:**
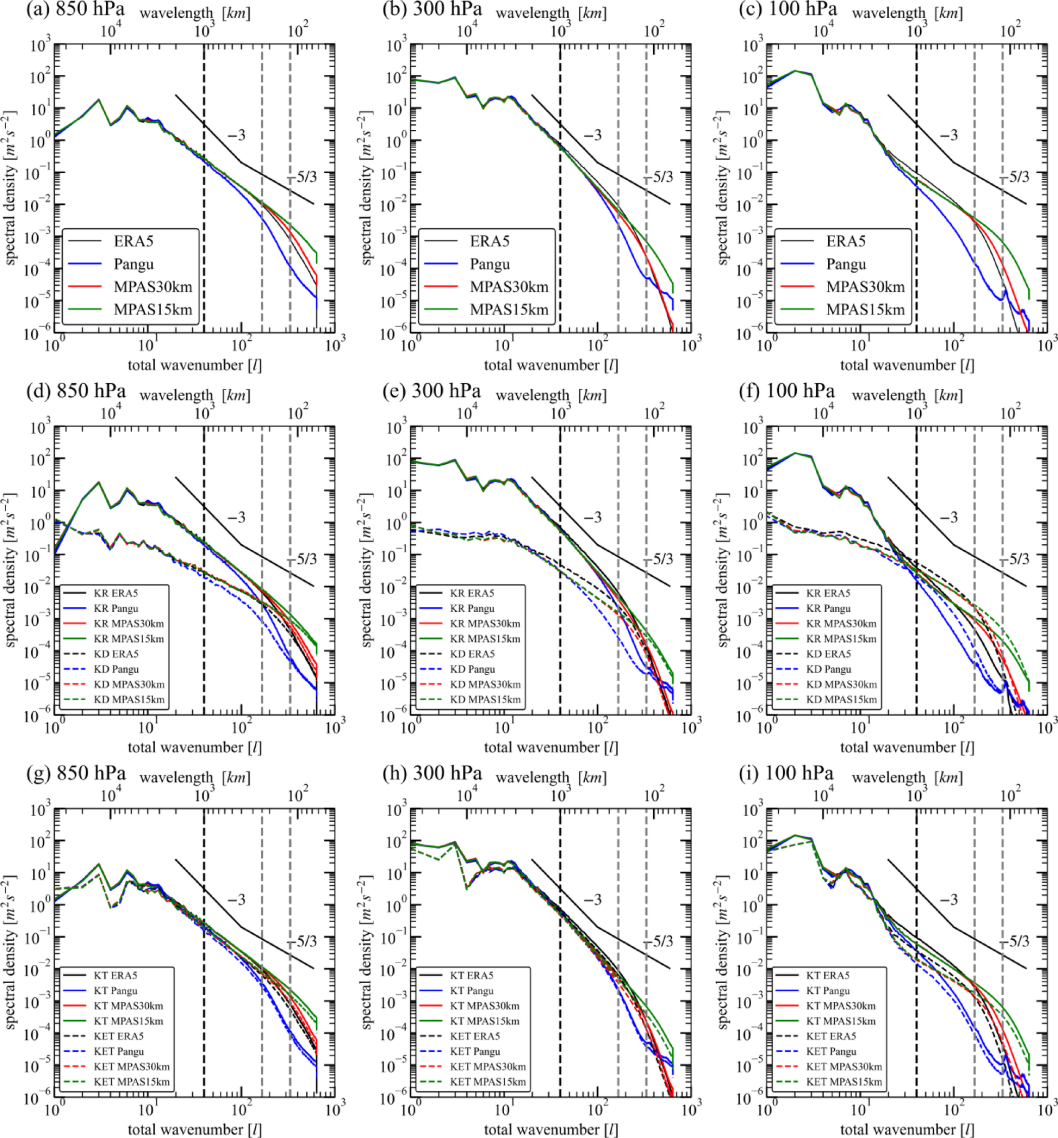
Wavenumber spectra of KE. (**a**–**c**) Horizontal kinetic energy spectra in ERA5 and different simulations at 850 hPa (left), 300 hPa (center) and at 100 hPa (right). (**d**–**f**) Rotational and divergent kinetic energy spectra. (**g**–**i**) Tropical and extratropical kinetic energy spectra. The − 3 and − 5/3 slopes are shown as black lines. The effective resolution of MPAS model is estimated as 8 times as the mesh space, i.e., 240 km and 120 km corresponding to the grid resolution of 30 km and 15 km, respectively. They are shown as vertical gray dashed lines. The vertical black dashed line represents the upper bound of the mesoscale range. The spectra are the mean of instantaneous KE spectra during 10 days.

The global spectra of horizontal kinetic energy (KH) are shown in Fig. 2a–c. At both 850 hPa and 300 hPa (Fig. 2a), the Pangu spectrum agrees with the ERA5 spectrum and the MPAS spectra at large scales ($\:l<40$). At mesoscales ($\:l\ge\:40$), the Pangu spectrum begins to be smaller than the ERA5 spectrum and MPAS spectra. This suggests that Pangu cannot simulate the same energy amplitude as the ERA5 at mesoscales, which is considered as the benchmark in this paper. At 300 hPa, the Pangu spectrum separate from the MPAS spectra at $\:l\approx\:80$ (about 500 km), and Pangu model simulates less mesoscale KE than the MPAS model from this scale (Fig. 2b). At 100 hPa, both MPAS and Pangu separate from ERA5 at $\:l\sim20$ (corresponding to wavelength about 2000 km), but Pangu misses the spectral transition characteristics (Fig. 2c). The results of different height layers show that the loss of energy at mesoscales of Pangu is not sensitive to the chosen vertical levels. Moreover, the higher the altitude, the larger the scale at which the Pangu spectrum starts to be smaller.

The rotational KE (KR) and divergent KE (KD) spectra based on Helmholtz decomposition are shown at Fig. 2d–f. At 850 hPa, the differences in the KR and KD spectra are mainly located at mesoscales (Fig. 2d). At 300 hPa, the KH is dominated by the KR until the dissipation range (wavelengths < 240 km), and the difference in the KD spectra is also substantial at large scales. At $\:l=100$, the Pangu KD spectrum is 0.25 of the ERA5 KD spectrum, while the Pangu KR spectrum is 0.56 times the ERA5 KR spectrum. This indicates that the difference in KD is relatively larger than that in KR. At 100 hPa, the KR spectrum intersects with the KD spectrum at $\:l\approx\:30$. Thus, the KD dominates the KH at mesoscales. At $\:l=100$, the Pangu KR spectrum is 0.126 times that of ERA5, and the Pangu KD spectrum is 0.148 times that of ERA5. The results show that compared with 300 hPa, the consistency of mesoscale KR and KD spectra between Pangu model and ERA5 at 100 hPa is poorer.

The KH spectra focused on the tropical region are calculated by multiplying the global field in grid-point space by $\:{cos}^{30}\left({\varphi\:}^{2}\right)$^30^. The extratropical spectra are calculated by multiplying the global field in grid-point space by 1-$\:{cos}^{30}\left({\varphi\:}^{2}\right)$. As shown in Fig. 2g–i, the spectra of the tropical KH (KT) are systematically stronger than those of the extratropical KH (KET) across all levels in both ERA5 and the models Pangu and MPAS. This contrast is mostly pronounced at 100 hPa (Fig. 2i) below 3000 km, where the KT energy density is approximately half an order of magnitude higher than KET. At $\:l>20$, Pangu shows a more abrupt drop in energy density of both KT and KET compared to MPAS (Fig. 2g–i). The results show that Pangu more significantly underestimates mesoscale kinetic energy in both the tropics and extratropics, especially in the lower stratosphere.

These KE spectra show that the Pangu significantly underestimate the atmospheric mesoscale energy. The performance of the KE spectra indicates that the effective resolution of the Pangu model is much lower than that of the ERA5, even lower than that of the MPAS model with similar nominal resolution.

**Fig. 3:**
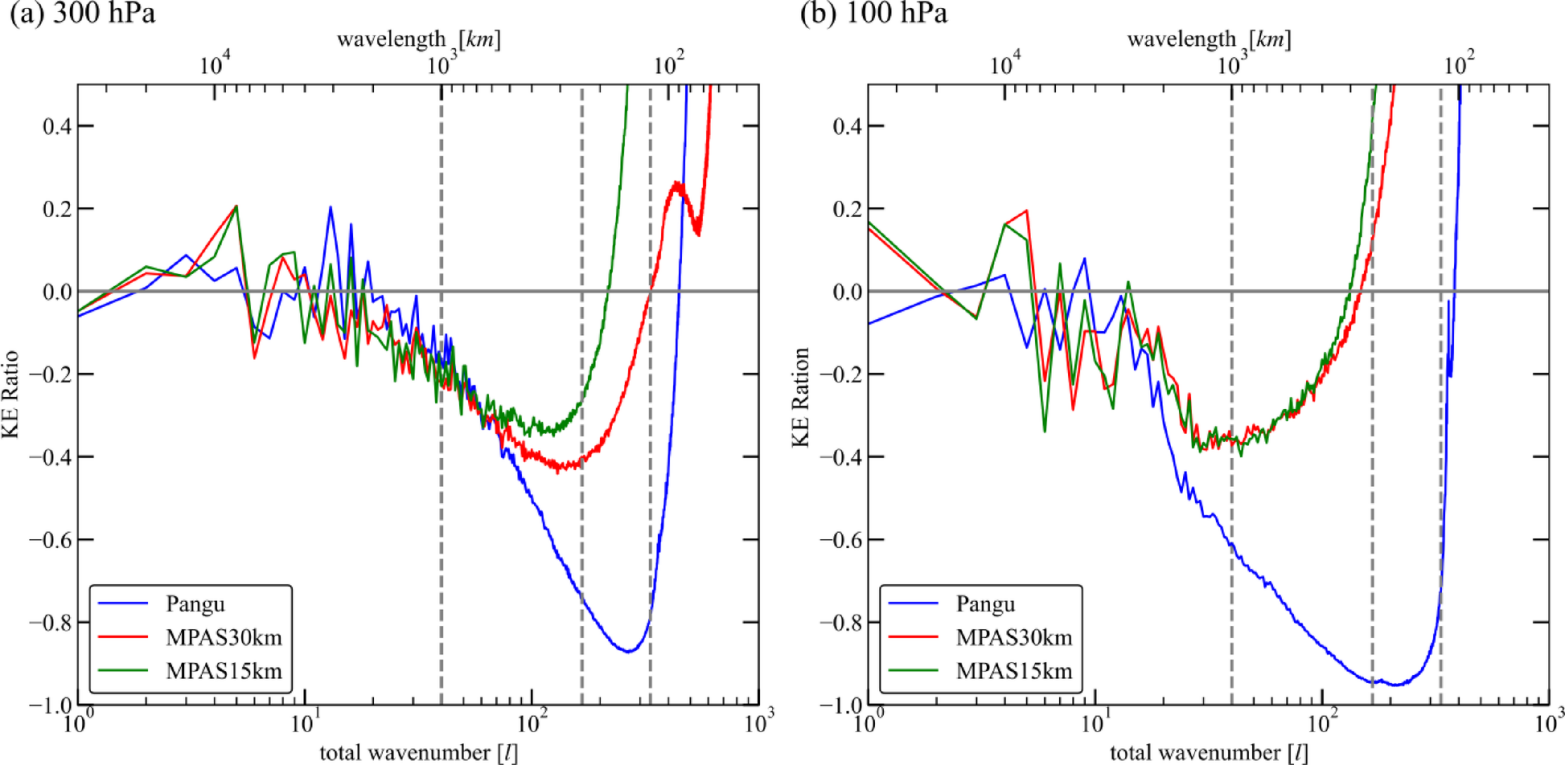
The relative differences in KH spectra between ERA5 and other simulations. (**a**) At 300 hPa. (**b**) at 100 hPa.

The relative differences, calculated by $\:(Model-ERA5)/ERA5$, are further shown in Fig. 3. At 300 hPa, the amplitude of relative difference of Pangu is smaller than 0.2 at large scales. At mesoscales, the relative differences exceed − 0.2, and the Pangu has a larger deviation from ERA5 than MPAS. It can be also seen that the higher the resolution of MPAS, the smaller the deviation from ERA5. At 100 hPa, the relative error of Pangu is larger than that at 300 hPa, with significant deviations starting from $\:l\approx\:20$. An obvious feature is that as scale decreases, this relative deviation rapidly increases. In addition, the differences between the two MPAS simulations with different resolutions are relatively small. This is a significant difference between the Pangu model and numerical models. At small scales, the expansion of relative difference is caused by the rapid attenuation of ERA5, possibly due to the numerical dissipation.

### Diagnosis of KH spectral budget

**Fig. 4:**
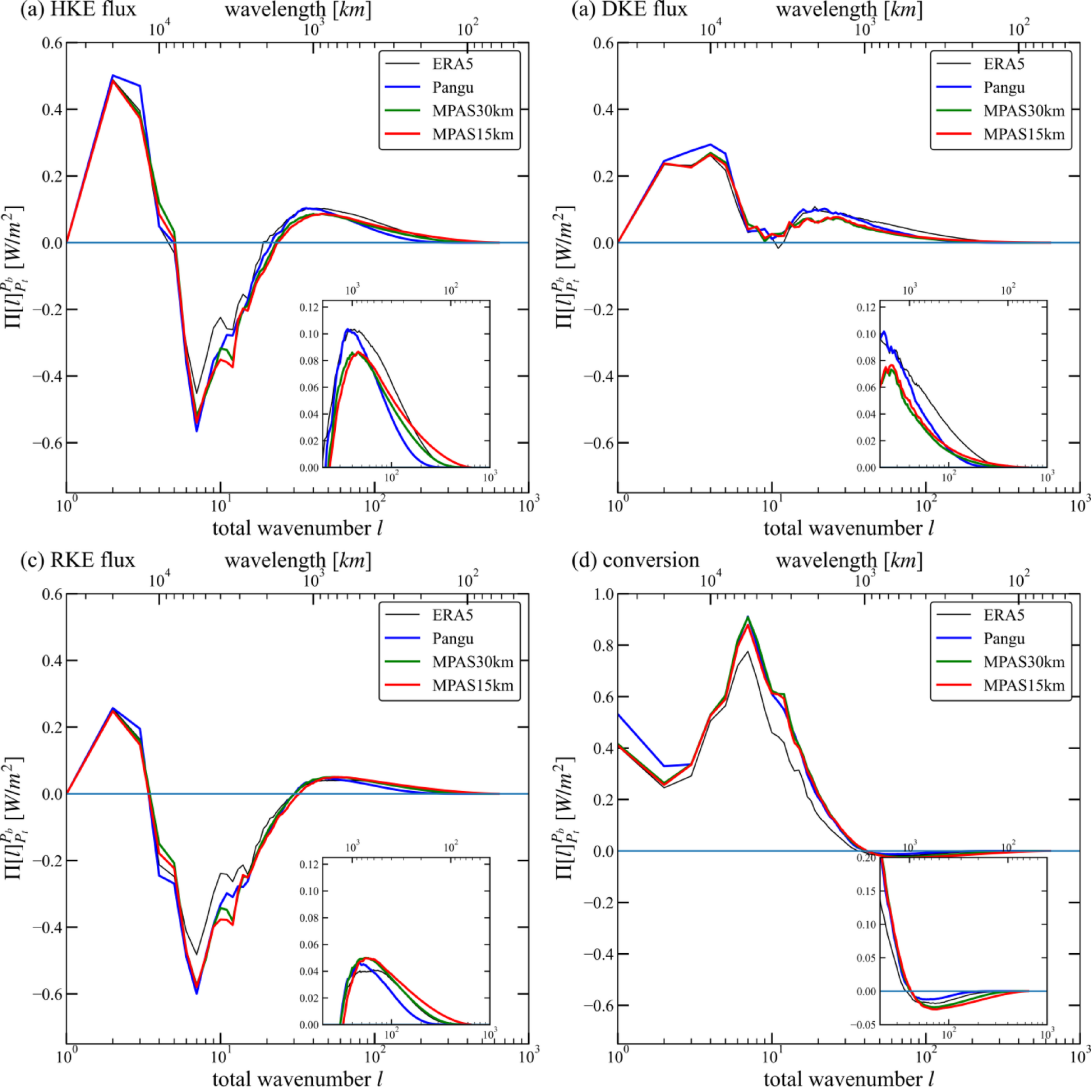
Spectral budget terms of ERA5, Pangu and MPAS. (**a**) KH flux vertically integrated from 400 to 250 hPa. (**b**) KD flux. (**c**) KR flux. (**d**) Cumulative conversion from KD to KR. The inset is an expanded view of the mesoscale subrange ($\:l\ge\:20$).

To follow differences in KH spectra in the upper troposphere, Fig. 4 shows the KH, KR, and KD spectral fluxes and conversion from KD to KR vertically integrated from 400 to 250 hPa in the dataset and models based on Eqs. (8–10). The comparison of spectral fluxes helps to illustrate the energy cycle at different scales and can provide more insights into the dynamics of the differences. Energy is deposited at the negative slope of each spectral flux curve (e.g., $\:d{\Pi\:}\left[l\right]/dl<0$) and removed at the positive slope; besides, the energy cascade is downscale at $\:{\Pi\:}\left[l\right]>0$ and upscale at $\:{\Pi\:}\left[l\right]<0$^28,31–33^.

Although the spectral flux curves are similar overall, there are some differences (Fig. 4). At large scales ($\:7\:<\:l\:<\:20$), the Pangu and MPAS exhibit stronger upscale energy cascades than ERA5, but at $\:l<7$, a downscale cascade is observed. The minimum value of the upscale KH spectral flux representing the intensity of cascade is -0.565 $\:W{m}^{-2}$ in Pangu, -0.520 $\:W{m}^{-2}$ in MPAS30, -0.540 $\:W{m}^{-2}$ in MPAS15, while it is -0.452 $\:W{m}^{-2}$ in ERA5 (Fig. 4a). The difference between models and ERA5 is mainly due to the rate of conversion from the KD to the KR (Fig. 4d), which energizes the KR upscale transfer. The stronger KR spectral flux corresponds to the stronger conversion from the KD to the KR in Pangu and MPAS. Since the upscale energy cascade is mainly powered by baroclinic instability^28^, this indicates stronger baroclinic instability described in these models than in ERA5. It also means that the Pangu simulation is the most consistent with the large-scale quasi-geostrophic features.

At $\:l\approx\:40$, the downscale cascade intensity (maximum) of KH calculated by the Pangu and ERA5 is almost the same, which is higher than that of the two MPAS experiments (Fig. 4a). It can be obviously seen that the difference in KH spectral flux is mainly due to the KD downscale cascade (Fig. 4b), while the KR downscale cascade intensity of the four groups was relatively close (Fig. 4c). The smaller mesoscale part ($\:l\ge\:100$) shows some significant differences in KH spectral flux. At $\:l=100$, the downscale KE spectral flux is 0.038 $\:W{m}^{-2}$ in Pangu, while it is 0.064 $\:W{m}^{-2}$ in ERA5. This indicates that less energy is transferred to mesoscales in Pangu, which is consistent with the smaller intensity of kinetic energy spectra. For the KD downscale cascade, both the Pangu model and the MPAS model are smaller than ERA5. It can also be seen that at these scales, the MPAS model exhibits more conversion of KR to KD.

At $\:l\ge\:200$, the three spectral fluxes and cumulative conversion are very small in Pangu. In contrast, the terms of the MPAS15 in this range are greater or smaller than 0. This shows that the higher the horizontal resolution of the physics-based model, the more converted energy can be simulated at smaller scales, that is, the stronger the ability to describe the multiscale interactions between rotational and divergent modes. Combined with the characteristics of the KE spectra, the performance of the Pangu model shows that it has the lowest effective resolution among the dataset and models.

### Spectrum of pressure vertical velocity

**Fig. 5:**
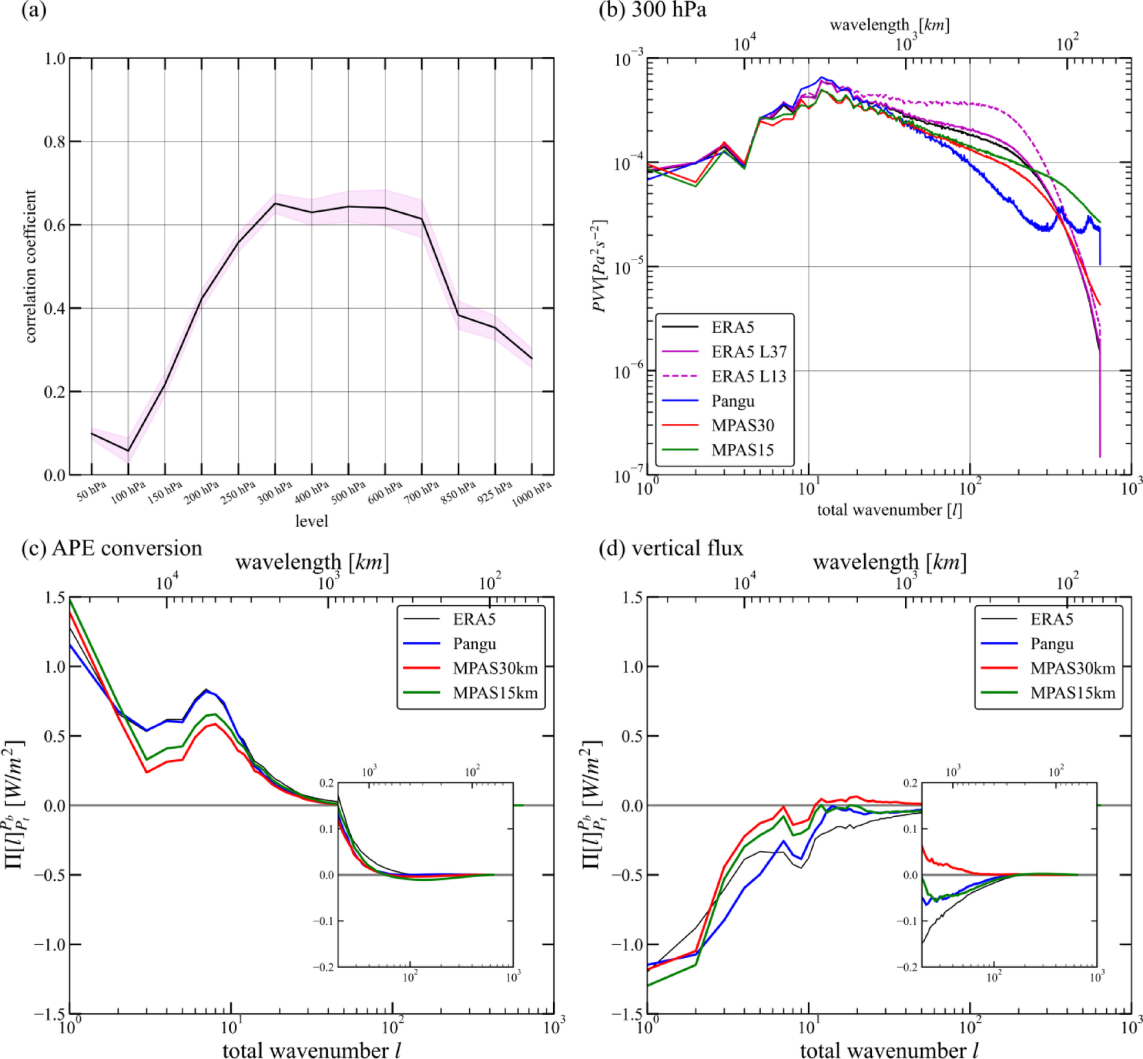
The pressure vertical velocity $\:\omega\:$ and related spectral budget terms. (**a**) The mean linear correlation coefficients between the diagnosed $\:\omega\:$ from partial ERA5 with the same 13 pressure levels as Pangu and the original ERA5 $\:\omega\:$ at each level. The purple shadow represents a range of positive and negative standard deviations. (**b**) The spectra of the original ERA5 $\:\omega\:$ are represented by a black line. ERA5 L37 (magenta line) represents using all the 37 levels to diagnose $\:\omega\:$. ERA5 L13 (meshed magenta line) represents using the same levels as Pangu to diagnose $\:\omega\:$. The spectrum of Pangu $\:\omega\:$ are represented by a blue line. The spectrum of MPAS30 $\:\omega\:$ is red. The spectrum of MPAS15 $\:\omega\:$ is green. They are shown at 300 hPa. (**c**) The accumulated APE conversion from 400 to 250 hPa. (**d**) The accumulated vertical flux from 400 to 250 hPa.

The pressure vertical velocity $\:\omega\:$ of Pangu is diagnosed with horizontal wind by Eq. (15). Prior to this, the accuracy of such diagnosis is first assessed with ERA5, with focus on its sensitivity to the selected pressure levels: the default 37 pressure levels (L37) and the same 13 pressure levels as Pangu (L13). Note that the original ERA5 $\:\omega\:$ is computed from horizontal winds based on the mass continuity equation in hybrid-sigma vertical coordinate with 137 levels. The correlation coefficient between the diagnosed $\:\omega\:$ using the ERA5 L13 and the original ERA5 $\:\omega\:$ is displayed in Fig. 5a. The correlation coefficients are calculated using the whole grid point at each level. The correlation coefficients exceed 0.6 from 300 hPa to 700 hPa, better than other levels.

Figure 5b shows the variance spectra of $\:\omega\:$ at 300 hPa. These spectra are very close to the ERA5 $\:\omega\:$ spectrum at $\:l\le\:20$. This indicates that this diagnosis has a certain degree of reliability within this scale range (Fig. 5b). In this scale range, Pangu’s vertical velocity spectrum is closest to ERA5, slightly higher than the two MPAS simulations. However, the spectrum of ERA5 L37 $\:\omega\:$ has a larger amplitude than that of ERA5 at smaller scales ($\:30\:<\:l\:<\:300$). This indicates that the coarser vertical resolution can increase the variance of vertical motion. This is further proven by the ERA5 L13 spectra with larger variance at smaller scales. The results indicate that the reduction of interpolation layers mainly affects the amplitude of spectra of $\:\omega\:$ at scales below 2000 km. The Pangu $\:\omega\:$ has a smaller variance at these scales. Note that the vertical grid of Pangu is only 13, and coarser than that of ERA5. Therefore, it is reasonable to infer that the Pangu model grossly underestimates the vertical motion at mesoscale scales. Note that the two peaks around $\:l\sim300\:$and $\:l\sim500$ in the Pangu model are likely model artifacts and do not represent physically meaningful phenomena.

For the spectral budget, the accumulated available potential energy (APE) conversion term (Fig. 5c) and the vertical flux term (Fig. 5d) are closely related to vertical motion based on Eqs. (13–14). The former represents the conversion of APE caused by temperature meridional gradient to KD through baroclinic instability, while the latter represent the vertical transport of KE and geopotential. The APE conversion term of Pangu is the closest to ERA5, showing stronger APE conversion to KD than MPAS at large scales. At mesoscales, the vertical fluxes of Pangu and MPAS15 remain negative forcing, while MPAS30 exhibits positive forcing. This once again demonstrates the accuracy of Pangu in large-scale simulations.

## Discussion

The main objective of this study is to examine the characteristics of atmospheric KE spectra generated by data-driven models. We conducted a medium-range (10-days) forecasting experiment using the Pangu model and MPAS model with same initial field. Based on the two common metrics to evaluate the prediction performance, i.e., latitude-weighted RMSE and ACC, the results show that the AI model has better short to medium-range forecasting skill compared to numerical model. The KE spectra of ERA5 reanalysis, Pangu simulation, and MPAS simulations are consistent at large scales. However, a noticeable reduction of energy at mesoscales is observed in Pangu compared to the ERA5 reanalysis and MPAS models in the upper troposphere and lower stratosphere. The difference in the KH spectrum is dominated by the KR spectrum in the upper troposphere, and dominated by the KD spectrum in the lower stratosphere. In the upper troposphere, the Pangu model underestimates KH in both the tropics and extratropics. In the lower stratosphere, the differences between the Pangu model and other data come mainly from the tropics. The relative difference between models and ERA5 shows that the Pangu model has the smallest deviation from ERA5 at large scales, but the deviation significantly increases with increasing height and decreasing scale. These features prove that the Pangu model owns the lowest effective resolution compared to the physics-based model with the close nominal resolution, which is around 1000 km in the upper troposphere and lower stratosphere. It is important to note that the closer agreement of Pangu’s kinetic energy spectra with ERA5 at large scales is likely due to the fact that Pangu was trained using ERA5 data. This does not necessarily imply that Pangu’s performance is superior to any physics-based models in representing the true atmospheric dynamics, just that the AI-based approach is capable of appropriately learning the relevant correlations responsible for large-scale KE spectra.

The KR and KD spectral budget show that the Pangu simulation, the MPAS simulations, and the ERA5 reanalysis presents similar characteristics, but there are two main differences in energy cascade. Firstly, compared with the ERA5, both the Pangu model and the MPAS model have stronger upscale KH cascades at $\:7\:<\:l\:<\:20$. The difference in the KH cascade is dominated by KR cascade. The stronger KR spectral flux energized by the stronger conversion from KD to KR in Pangu, indicating a stronger baroclinic instability. Secondly, at$\:\:l\:>\:20$, the intensity of downscale cascade of the Pangu model is close to the ERA5, which is dominated by the KD. However, the Pangu simulation has the least energy deposited from downscale cascade at smaller mesoscale ($\:l>80$), which is one of the main reasons for the smallest intensity of the KE spectra at mesoscales.

The vertical motion closely related to the convection and gravity wave flux can be diagnosed by divergence. The results show that the weak KD leads to weak vertical motion. The results show that the intensity of the Pangu $\:\omega\:$ exceeds that of MPAS at large scales but rapidly decreases at mesoscales. This indicates that the Pangu model underestimates the vertical motion and has a weak ability to describe the mesoscale convergence and divergence motion. Vertical motion plays a crucial role in the KE spectra and spectral budget, as it directly affects the vertical flux and APE conversion^34^. The APE conversion term and the vertical flux term of Pangu are closest to those of ERA5, showing stronger APE conversion to divergent kinetic energy and net energy outflow at large scales. At mesoscales, the vertical fluxes of Pangu and MPAS15 remain negative contribution, while MPAS30 exhibits positive contribution.

## Conclusion

Although the Pangu model has the lowest effective resolution, it achieves the highest prediction accuracy based on RMSE and ACC evaluations. However, its inability to reasonably simulate mesoscale energy limits the model’s reliable prediction of mesoscale systems, especially for rapidly developing extreme weather systems. The advantage of the Pangu model in simulating large-scale vertical motion enables it to better predict the baroclinic instability and Rossby waves but its ability to describe energy at mesoscale is insufficient. For data-driven AI approaches, this may be due to the convolution operations focusing on capturing large-scale information, or the design of the loss function, resulting in the model training process filtering out some mesoscale information. Given the importance of vertical motion, it is also recommended to consider vertical motion as a variable in AI-model training.

In this study, the Pangu model shows closer agreement with ERA5 at large scales but underestimates mesoscale kinetic energy compared to MPAS. These results highlight the limitations of the Pangu model in simulating mesoscale energy. However, it is important to note that the conclusions drawn in this study are specific to the Pangu and MPAS models used here. Future work should explore additional model configurations and observational datasets to further validate our findings. Additionally, the use of other physics-based models with different configurations and parameterizations could provide a more comprehensive understanding of the differences between AI-based and physics-based models.

## Data Availability

ERA5 reanalysis data is available via the Copernicus Climate Data Store (https://cds.climate.copernicus.eu/). The “Pangu Weather” model is available on GitHub (https://github.com/198808xc/Pangu-Weather). The MPAS model is available on GitHub (https://github.com/MPAS-Dev/MPAS-Model/releases/tag/v7.3). The data that support the findings of this study have been deposited in the figshare platform via the DOI 10.6084/m9.figshare.27262035. For access to the original model outputs, please contact the corresponding author, Jun Peng, at pengjun@nudt.edu.cn.

## References

[1] Pathak, J. et al. FourCastNet: A Global Data-driven High-resolution Weather Model using Adaptive Fourier Neural Operators. Preprint at (2022). http://arxiv.org/abs/2202.11214

[2] Bi, K. et al. Accurate medium-range global weather forecasting with 3D neural networks. *Nature* **619**, 533–538 (2023). 37407823 10.1038/s41586-023-06185-3 PMC10356604

[3] Chen, L. et al. FuXi: a cascade machine learning forecasting system for 15-day global weather forecast. *Npj Clim. Atmos. Sci.* **6**, 190 (2023).

[4] Charlton-Perez, A. J. et al. Do AI models produce better weather forecasts than physics-based models? A quantitative evaluation case study of storm Ciarán. *Npj Clim. Atmos. Sci.* **7**, 93 (2024).

[5] Selz, T. & Craig, G. C. Can artificial intelligence-based weather prediction models simulate the butterfly effect? *Geophys. Res. Lett.* **50**, e2023GL105747 (2023).

[6] Storer, B. A., Buzzicotti, M., Khatri, H., Griffies, S. M. & Aluie, H. Global energy spectrum of the general oceanic circulation. *Nat. Commun.* **13**, 5314 (2022). 36085140 10.1038/s41467-022-33031-3 PMC9463453

[7] Nastrom, G. D., Gage, K. S. & Jasperson, W. H. Kinetic energy spectrum of large-and mesoscale atmospheric processes. *Nature* **310**, 36–38 (1984).

[8] Nastrom, G. D. & Gage, K. S. A climatology of atmospheric wavenumber spectra of wind and temperature observed by commercial aircraft. *J. Atmos. Sci.* **42**, 950–960 (1985).

[9] Skamarock, W. C., Park, S. H., Klemp, J. B. & Snyder, C. Atmospheric kinetic energy spectra from global High-Resolution nonhydrostatic simulations. *J. Atmos. Sci.* **71**, 4369–4381 (2014).

[10] Waite, M. L. Untangling waves and vortices in the atmospheric kinetic energy spectra. *J. Fluid Mech.* **888**, F1 (2020).

[11] Wang, J. W. A. & Sardeshmukh, P. D. Inconsistent global kinetic energy spectra in reanalyses and models. *J. Atmos. Sci.* **78**, 2589–2603 (2021).

[12] Kouhen, S., Storer, B. A., Aluie, H., Marshall, D. P. & Christensen, H. M. Convective and Orographic Origins of the Mesoscale Kinetic Energy Spectrum. *Geophys. Res. Lett.* 51, e2024GL110804 (2024).

[13] Skamarock, W. C. Evaluating mesoscale NWP models using kinetic energy spectra. *Mon Wea Rev.* **132**, 3019–3032 (2004).

[14] Brune, S. & Becker, E. Indications of stratified turbulence in a mechanistic GCM. *J. Atmos. Sci.* **70**, 231–247 (2013).

[15] Li, Z., Peng, J. & Zhang, L. Impact of convective parameterizations on atmospheric mesoscale kinetic energy spectra in global High-Resolution simulations. *Geophys. Res. Lett.* **50**, e2023GL105513 (2023).

[16] Lorenz, E. N. The predictability of a flow which possesses many scales of motion. *Tellus* **21**, 289–307 (1969).

[17] Weyn, J. A. & Durran, D. R. The dependence of the predictability of mesoscale convective systems on the horizontal scale and amplitude of initial errors in idealized simulations. *J. Atmos. Sci.* **74**, 2191–2210 (2017).

[18] Hersbach, H. et al. The ERA5 global reanalysis. *Q. J. R Meteorol. Soc.* **146**, 1999–2049 (2020).

[19] Dosovitskiy, A. et al. An Image is Worth 16x16 Words: Transformers for Image Recognition at Scale. Preprint at (2021). http://arxiv.org/abs/2010.11929

[20] Klemp, J. B., Dudhia, J. & Hassiotis, A. D. An upper Gravity-Wave absorbing layer for NWP applications. *Mon Wea Rev.* **136**, 3987–4004 (2008).

[21] Zhang, C. & Wang, Y. Projected future changes of tropical cyclone activity over the Western North and South Pacific in a 20-km-Mesh regional climate model. *J. Clim.* **30**, 5923–5941 (2017).

[22] Hong, S. Y. & Lim, J. The WRF Single-Moment 6-Class microphysics scheme (WSM6). *Asia-Pac. J. Atmos. Sci.* (2006).

[23] Tewari, M. et al. 14.2A implementation and verification of the unified Noah land surface model in the Wrf model (2004).

[24] Hong, S. Y., Noh, Y. & Dudhia, J. A. New vertical diffusion package with an explicit treatment of entrainment processes. *Mon. Weather Rev.* **134**, 2318–2341 (2006).

[25] Janji, Z. I. Nonsingular Implementation of the Mellor-Yamada Level 2.5 Scheme in the NCEP Meso model. *Office note (National Centers for Environmental Prediction (U.S.)); 437* (2001).

[26] Iacono, M. J. et al. Radiative forcing by long-lived greenhouse gases: calculations with the AER radiative transfer models. *J. Geophys. Research: Atmos.* **113** (2008).

[27] Rasp, S. et al. WeatherBench: A benchmark data set for data-driven weather forecasting. *J. Adv. Model. Earth Syst.* **12**, e2020MS002203 (2020).

[28] Li, Z., Peng, J. & Zhang, L. Spectral budget of rotational and divergent kinetic energy in global analyses. *J. Atmos. Sci.* **80** (2023).

[29] Müller, S. K., Manzini, E., Giorgetta, M., Sato, K. & Nasuno, T. Convectively generated gravity waves in high resolution models of tropical dynamics. *J. Adv. Model. Earth Syst.* **10**, 2564–2588 (2018).

[30] Polichtchouk, I., Wedi, N. & Kim, Y. Resolved gravity waves in the tropical stratosphere: impact of horizontal resolution and deep convection parametrization. *Q. J. R Meteorol. Soc.* **148**, 233–251 (2022).

[31] Augier, P. & Lindborg, E. A. New formulation of the spectral energy budget of the atmosphere, with application to two High-Resolution general circulation models. *J. Atmos. Sci.* **70**, 2293–2308 (2013).

[32] Malardel, S. & Wedi, N. P. How does subgrid-scale parametrization influence nonlinear spectral energy fluxes in global NWP models? *J. Geophys. Res. Atmos.* **121**, 5395–5410 (2016).

[33] Wedi, N. P. et al. A baseline for global weather and climate simulations at 1 Km resolution. *J. Adv. Model. Earth Syst.* **12** (2020).

[34] Li, Z., Peng, J., Zhang, L. & Guan, J. Exploring the differences in kinetic energy spectra between the NCEP FNL and ERA5 datasets. *J. Atmos. Sci.* **81**, 363–380 (2024).

